# Supplementary material for: Integrating the Electronic Health Record Into Patient Encounters: An Introductory Standardized Patient Exercise for Preclinical Medical Students
Source: MedEdPORTAL. 2022 Jan 5;18:11209. doi: 10.15766/mep_2374-8265.11209 (PMC8727442; doi:10.15766/mep_2374-8265.11209)
Supplement: Supplementary file 1 — Door Charts.docxSimulated EHR.pptxSession Logistics.docxSP Scripts and Instructions.docxPreclass Student Notes.docxPerspectives From Faculty on EHR.mp4Faculty Instructions.docxEHR Skills - Good Example.mp4EHR Skills - Bad Example.mp4Learner Evaluation.docx [file mep_2374-8265.11209-s001.zip › J. Learner Evaluation.docx]

**Doctor-Patient Relationship (DPR) & the Electronic Health Record (EHR):**

**Learner Evaluation**

**Survey Purpose**

The following questions refer to your experience during the recent clinical skills class session, during which we introduced concepts about how the Electronic Health Record (EHR) impacts the doctor-patient relationship (DPR).

The purpose of this survey is to collect your feedback on the educational value of this lesson and its materials. Your responses on this survey will be used to improve how we provide this instruction for future medical students. Your instructors will not know whether you completed this survey or how you answered the questions.  Your identity will remain confidential, and your responses will have no impact on your course grades.

**Session Design and Content:**

***The following questions refer to the pre-class materials for this class session:***

Was the amount of time required to review pre-class materials manageable?

- Yes
- No

How effective were the pre-class student notes on the DPR and the EHR, for learning about how to navigate the DPR while interacting with the EHR?

- Extremely effective
- Quite effective
- Moderately effective
- Slightly effective
- Not at all effective

How effective was the conceptual framework, “HUMAN LEVEL” (included in pre-class materials), for learning about how to navigate the DPR while interacting with the EHR?

- Extremely effective
- Quite effective
- Moderately effective
- Slightly effective
- Not at all effective

How effective was the video, “Advice from Experienced Faculty on the EHR and the Practice of Medicine,” for learning about how to navigate the DPR while interacting with the EHR?

- Extremely effective
- Quite effective
- Moderately effective
- Slightly effective
- Not at all effective
- I did not review this optional resource.

***The following questions refer to the in-class standardized-patient (SP) activity during which students practiced conducting focused patient encounters for respiratory chief complaints while interacting with a simulated EHR:***

How effective were these SP encounters, for ***learning about how to navigate*** the DPR while interacting with the EHR?

- Extremely effective
- Quite effective
- Moderately effective
- Slightly effective
- Not at all effective

How effective were these SP encounters, for ***understanding the challenges*** of navigating the DPR while interacting with the EHR?

- Extremely effective
- Quite effective
- Moderately effective
- Slightly effective
- Not at all effective

Was the small-group format of this learning activity appropriate for the learning content presented?

- Yes
- No

Were the pace and duration of this learning activity appropriate for the learning content presented?

- Yes
- No

***The following questions refer to this class session, as a whole:***

How relevant was the content of this class session to your role as a future physician?

- Extremely relevant
- Quite relevant
- Moderately relevant
- Slightly relevant
- Not at all relevant

How effective was this class session for helping you to meet each of the following learning objectives?

| Demonstrate strategies for effectively maintaining the doctor-patient relationship while interacting with the electronic health record. | Extremely effective | Quite effective | Moderately effective | Slightly effective | Not effective at all |
| --- | --- | --- | --- | --- | --- |
| Verify and update data gathered from the electronic health record with the patient during the encounter. | Extremely effective | Quite effective | Moderately effective | Slightly effective | Not effective at all |

What was the overall effectiveness of this learning activity? (1 = poor; 10 = excellent): *<scale of 1 to 10>*

Which aspects of this activity were most effective? *<free text field>*

Which aspects of this activity could be improved? (Please provide specific suggestions): *<free text field>*
