# Supplementary material for: Integrating the Electronic Health Record Into Patient Encounters: An Introductory Standardized Patient Exercise for Preclinical Medical Students
Source: MedEdPORTAL. 2022 Jan 5;18:11209. doi: 10.15766/mep_2374-8265.11209 (PMC8727442; doi:10.15766/mep_2374-8265.11209)
Supplement: Supplementary file 1 — Door Charts.docxSimulated EHR.pptxSession Logistics.docxSP Scripts and Instructions.docxPreclass Student Notes.docxPerspectives From Faculty on EHR.mp4Faculty Instructions.docxEHR Skills - Good Example.mp4EHR Skills - Bad Example.mp4Learner Evaluation.docx [file mep_2374-8265.11209-s001.zip › A. Door Charts.docx]

**Patient name:** Fran Simon

**Age:** 56 years

**Chief complaint:** cough

**Vitals:**

Temperature: 99.6° F

Heart rate: 80 bpm

Respiratory rate: 14 bpm

Blood pressure: 136/84 mm Hg

O_2_ Sat: 92% on room air

**Patient name:** Pat Lopez

**Age:** 30 years

**Chief complaint:** shortness of breath

**Vitals:**

Temperature: 98.4° F

Heart rate: 95 bpm

Respiratory rate: 18 bpm

Blood pressure: 125/72 mm Hg

O_2_ Sat: 96% on room air

**Patient name:** Riley Yates

**Age:** 52 years

**Chief complaint:**  cough

**Vitals:**

Temperature: 100.7° F

Heart rate: 92 bpm

Respiratory rate: 12 bpm

Blood pressure: 115/78 mm Hg

O_2_ Sat: 93% on room air

**Patient name:** Peyton Tran

**Age:** 61 years

**Chief complaint:** shortness of breath

**Vitals:**

Temperature: 97.4° F

Heart rate: 74 bpm

Respiratory rate: 14 bpm

Blood pressure: 145/88 mm Hg

O_2_ Sat: 90% on room air
