# Supplementary material for: Integrating the Electronic Health Record Into Patient Encounters: An Introductory Standardized Patient Exercise for Preclinical Medical Students
Source: MedEdPORTAL. 2022 Jan 5;18:11209. doi: 10.15766/mep_2374-8265.11209 (PMC8727442; doi:10.15766/mep_2374-8265.11209)
Supplement: Supplementary file 1 — Door Charts.docxSimulated EHR.pptxSession Logistics.docxSP Scripts and Instructions.docxPreclass Student Notes.docxPerspectives From Faculty on EHR.mp4Faculty Instructions.docxEHR Skills - Good Example.mp4EHR Skills - Bad Example.mp4Learner Evaluation.docx [file mep_2374-8265.11209-s001.zip › C. Session Logistics.docx]

**Doctor-Patient Relationship (DPR) & the Electronic Health Record (EHR):**

**Session Logistics**

**Timetable, day of event:**

The event duration can be adjusted according to the learner group size, one’s space needs and exam room availability, and one’s desired number of cases. The following is an example rotation schedule for an event designed for 4 student small groups, using 4 rooms, and using all 4 cases. During each encounter (A – D), 20 minutes will be spent conducting the SP encounter with 10 minutes for feedback. We recommend 5 minutes between encounters to allow for the small groups to switch rooms and for students to briefly review the EHR and door chart.

| **Time** | **Activity** | | **Description** |
| --- | --- | --- | --- |
| 15 minutes | Small group review of pre-class assignments | | The instructor briefly reviews with students the important concepts from the pre-class assigned materials, as well as provides brief instructions to students on the logistics for the activity. |
| 5 minutes | Transition to first encounter | | Students move to their first assigned exam room. |
| 20 minutes | Encounter A | Patient encounter/data gathering: Student A | One student in each group interviews and examines the standardized patient (SP) in their assigned exam room (other students and faculty instructor observe). |
| 10 minutes |  | Small group debrief | Student interviewer rejoins the small group to debrief on their performance during the encounter. |
| 5 minutes | Transition to next encounter | | Students move to their next assigned exam room. |
| 20 minutes | Encounter B | Patient encounter/data gathering: Student B | One student in each group interviews and examines the standardized patient (SP) in their assigned exam room (other students and faculty instructor observe). |
| 10 minutes |  | Small group debrief | Student interviewer rejoins the small group to debrief on their performance during the encounter. |
| 5 minutes | Transition to next encounter | | Students move to their next assigned exam room. |
| 20 minutes | Encounter C | Patient encounter/data gathering: Student C | One student in each group interviews and examines the standardized patient (SP) in their assigned exam room (other students and faculty instructor observe). |
| 10 minutes |  | Small group debrief | Student interviewer rejoins the small group to debrief on their performance during the encounter. |
| 5 minutes | Transition to next encounter | | Students move to their next assigned exam room. |
| 20 minutes | Encounter D | Patient encounter/data gathering: Student D | One student in each group interviews and examines the standardized patient (SP) in their assigned exam room (other students and faculty instructor observe). |
| 10 minutes |  | Small group debrief | Student interviewer rejoins the small group to debrief on their performance during the encounter. |

**Student group rotation schedule**

| **Encounter** | **Time** | **Case 1** | **Case 2** | **Case 3** | **Case 4** |
| --- | --- | --- | --- | --- | --- |
|  |  | Room 1 | Room 2 | Room 3 | Room 4 |
| Start | 5 minutes  (move to first encounter) | Students move to their first assigned exam room and review EHR and door chart. | | | |
| A | 20 minutes  (SP encounter) | Group 1 | Group 2 | Group 3 | Group 4 |
|  | 10 minutes  (debrief) |  |  |  |  |
|  | 5 minutes  (transition to next encounter) | Students move to their next assigned exam room and review EHR and door chart. | | | |
| B | 20 minutes  (SP encounter) | Group4 | Group 1 | Group 2 | Group 3 |
|  | 10 minutes  (debrief) |  |  |  |  |
|  | 5 minutes  (transition to next encounter) | Students move to their next assigned exam room and review EHR and door chart. | | | |
| C | 20 minutes  (SP encounter) | Group 3 | Group 4 | Group 1 | Group 2 |
|  | 10 minutes  (debrief) |  |  |  |  |
|  | 5 minutes  (transition to next encounter) | Students move to their next assigned exam room and review EHR and door chart. | | | |
| D | 20 minutes  (SP encounter) | Group 2 | Group 3 | Group 4 | Group 1 |
|  | 10 minutes  (debrief) |  |  |  |  |

*The following are examples of student schedule slips for 2 groups, based on the above rotation schedule (provided to students prior to event):*

| **Small group 1** | Encounter A | Encounter B | Encounter C | Encounter D |
| --- | --- | --- | --- | --- |
| Student A | Room 1  (Case 1) | Room 2  (Case 2) | Room 3  (Case 3) | Room 4  (Case 4) |
| Student B |  |  |  |  |
| Student C |  |  |  |  |
| Student D |  |  |  |  |
|  |  |  |  |  |
| **Small group 2** |  |  |  |  |
| Student E | Room 2  (Case 2) | Room 3  (Case 3) | Room 4  (Case 4) | Room 1  (Case 1) |
| Student F |  |  |  |  |
| Student G |  |  |  |  |
| Student H |  |  |  |  |

Our simulation center has the capability for overhead intercom announcements, so we instructed students and faculty to listen for these announcements throughout the activity, to facilitate staying on schedule.
