# Supplementary material for: Integrating the Electronic Health Record Into Patient Encounters: An Introductory Standardized Patient Exercise for Preclinical Medical Students
Source: MedEdPORTAL. 2022 Jan 5;18:11209. doi: 10.15766/mep_2374-8265.11209 (PMC8727442; doi:10.15766/mep_2374-8265.11209)
Supplement: Supplementary file 1 — Door Charts.docxSimulated EHR.pptxSession Logistics.docxSP Scripts and Instructions.docxPreclass Student Notes.docxPerspectives From Faculty on EHR.mp4Faculty Instructions.docxEHR Skills - Good Example.mp4EHR Skills - Bad Example.mp4Learner Evaluation.docx [file mep_2374-8265.11209-s001.zip › D. SP Scripts and Instructions.docx]

Electronic Health Record Exercise:

Standardized Patient Instructions

Lesson Overview

The objective of this exercise is to give students an opportunity to practice using patient-centered communication skills while simultaneously interacting with the patient’s electronic health record on the computer in the exam room. During this exercise, you will be playing the role of one of the patients in an outpatient clinic who is presenting with either “cough,” “shortness of breath,” or both of these symptoms.

Logistics

This exercise consists of 4 patient cases, and each SP participating in this exercise will play 1 of these cases. SPs will remain in the same, assigned exam room for the duration of this exercise.

Students will rotate among a series of rooms during which they will practice interviewing and examining you as the patients in this exercise. By the end of the exercise, a student from each small group will have interacted with SPs portraying each of the 4 cases, and you will have interacted with a total of 4 different students.

For each patient encounter, one student will lead the encounter, while the other students and the faculty observe the interaction.* Each patient encounter will be allotted 20 minutes, total, for the history taking and physical exam. After 20 minutes has passed, the faculty and students will spend the next 10 minutes reflecting on the interaction and providing feedback for the student who led the encounter.**

At the end of the feedback period, students and faculty will transition to the next room (after which you will have a new student to interact with, for your assigned case). During the transition period, please reset the computer screen by clicking the “overview” link in the simulated electronic medical record *(please do not navigate to a different webpage).*

This label on each screen indicates the patient whose “medical record” is displayed on the computer. Make sure this label matches the patient case you are portraying. If you see the wrong name here, click the house icon at the top right of the screen, and then select your case name from the patient list.

** The other students and faculty may be in the room with you (if space permits) or may be watching the encounter remotely from another location.*

*** The feedback session may occur in your exam room (if space permits) or in another location.*

Preparation for this encounter

Please review the details of your assigned case. We will review these cases as a group at the SP training session for this event. Please bring any questions you have about your case to this meeting.

Students will need to examine your chest area during this part of the exam, so please dress accordingly so that students can easily examine your chest area during this part of the exam. You will be given a hospital gown and a drape to wear over your underclothes.

Domains and Learning Objectives:

Domains: Check all that apply

- Professionalism

X	Communication and Interpersonal skills

X	Medical History

X	Physical exam

- Shared Decision Making
- Patient Education
- Clinical Reasoning
- Documentation
- Handoff
- Presentation
- Other:

Type and level of learner: First or second year medical students

Case Objectives: please list specific objectives for each of the domains you have checked above:

1. Practice communication strategies to effectively navigate the doctor-patient relationship while interacting with the electronic health record (EHR).
2. Verify information from a simulated EHR while obtaining history from a standardized patient during a simulated clinical encounter.
3. Perform additional, focused history taking based on data verified from the EHR.

Physical exam cards

Each case has 1 or more physical exam findings cards, which you will be expected to give to a student if they perform the physical exam maneuver that would reveal the information on each card. Please review your specific script for details about the card(s) associated with the physical exam portion of your encounter. During the encounter, please hide the PE cards from view, but keep them close by.

Please give each PE card immediately after the student performs the exam maneuver that prompts it, according to the instructions provided in your script; please do not wait until the end of the physical exam to provide these cards to students, if they performed the applicable maneuvers to prompt them.

Collect all PE cards from students before they leave the exam room. Students may write down the information from each PE card while they are in the room, but they must give the cards back to you before they leave.

Authors:

Primary Case Author: Donna Williams, MD

Secondary Case Author: Jennifer Jackson, MD

Standardized Patient Educators: Erin Haynes, Donna Williams, MD, and Jennifer Jackson, MD

*MedEdPORTAL* Standardized Patient Case Development Tool (Cases 1 - 4)

**Case 1:** New diagnosis of lung cancer

Patient Name: Fran Simon

Chief Complaint: “cough”

Most likely Diagnosis and Differential with rationale from history and/or physical exam: This patient has a new diagnosis of lung cancer. Lung cancer is most likely in this case duet to the patient’s demographics, smoking history, slowly progressive cough with bloody sputum, and physical exam findings including the lymphadenopathy and presence of a pleural effusion. Appropriate evaluation in this case would include ordering a complete blood count, complete metabolic panel, and CT of the chest with contrast.

| SETTING: outpatient, in patient, ED, home, nursing home, rehab, group etc. | Primary care clinic |
| --- | --- |
| PATIENT PROFILE: | |
| Age range | 50-60 years |
| Religious/spiritual background | Any |
| Sex (e.g., male, female, intersex, transwoman, transman) | Any |
| Sexual Orientation (e.g., heterosexual, lesbian, gay, bisexual, pansexual, queer, asexual) | Any |
| Gender expression (e.g., man, woman, gender queer) | Any |
| Race/ethnicity: | Any |
| Physical description (e.g., BMI, height range) | Average BMI |
| Physical limitations | None |
| Patient appearance (e.g., disheveled, hospital gown, business casual, casual) | Hospital gown |
| Moulage + location (e.g., none, bruises, scars, body piercing, tattoos) | None |
| Affect (e.g., pleasant, cooperative) | Pleasant, cooperative |
| Family group (e.g., who is family, who they live with) | I live at home with my spouse and 2 children. |
| Education | Graduated high school and completed some college courses |
| Level of health literacy | Average |
| Employment, if any - present and past, noting any current stresses | I work as a furniture salesperson. |
| Home/homeless - type of dwelling, number of stories, owned or rented | I live in a single-family home with my spouse and 2 children. |
| Financial situation- any current stresses | No current stresses. |
| Insurance Status (e.g., un/under/insured, public/private, HMO/PPO) | I am insured through my employer. |
| Habits (i.e., diet, exercise, caffeine, smoking, alcohol, drugs) | Diet: I try to eat healthy by eating a mix of protein and vegetables with few starches.  Exercise: I do not exercise routinely.  Tobacco: I started smoking cigarettes at age 15 and have smoked 1.5 packs per day since then.  Alcohol: None  Drugs: None |
| Activities (i.e., hobbies, sports, clubs, friends) | I enjoy watching action movies on Netflix. |
| Typical day - what is the usual daily routine | On a typical day, I wake up at 7am and get ready for work, which starts at 9am. After work, I come home, eat dinner with my family, and watch television or movies. |

| CASE INFORMATION | |
| --- | --- |
| Chief Concern: What the patient will say when greeted by the student. The patient’s primary reason for seeking medical care often stated in his/own words. | I have this cough that’s really bothering me, and I just don't feel well. |
| Additional Concerns: Other, if any, concerns the patient has today (i.e., symptoms, requests, expectations, etc.) that will become part of set agenda. | Fatigue, weight loss |
| THE PATIENT STORY: The SP will be asked to tell their symptom story and the personal and emotion impact for each of their concerns. You will want to write this is the patient voice. The symptom story should be able to answer this question: “Tell me more about [chief concern/additional concern], starting at the beginning and bringing me up to now.”  The personal context should be able to answer questions concerning the broader personal/psychosocial context of symptoms, especially the patient beliefs/attributions.  The emotional context should be able to ask how are you doing with this, how does this make you feel, how has this affected you emotionally? IMPACT: How has this affected your life? How has this been for your family? | I’ve had a cough for a long time due to my lung disease, but it seems to be getting worse. I don’t know what changed, but in the last couple months, the cough seems to be gradually worsening, day by day.  On top of that, I’ve just felt more tired and I think I’m losing weight, but it’s not like I’m trying to.  Personal/emotional context: I don’t know why the cough is worse or why I’m coughing up some blood now, but I’m worried that it could be cancer or something else that’s serious.  Impact (incl. associated symptoms): Over the past few months, I seem more tired than I used to. I don’t have any specific weakness, I just feel like I can’t do as much as I used to. But I am still able to go to work and go about my day. |
| HISTORY OF PRESENT ILLNESS: Although some of the HPI will be given in the patient’s symptom story, the learners will expand the story during the direct question section. Below describe the detailed history, usually about the chief concern, which the student must develop in order to make a useful assessment of the problem: | |
|  | |
| Onset (when; gradual or sudden) | I’ve had a cough for a long time, but it seems to be gradually getting worse over the past couple months. |
| Setting (what was going on or where was patient when symptoms first noticed?) | I wasn’t doing anything out of the ordinary when it started getting worse. |
| Duration (how long) | About 2 months |
| Time relationships (frequency, constant or intermittent) | The cough is intermittent throughout the day, although it seems to occur less as the day goes on. |
| Location | N/A |
| Radiation | N/A |
| Quality | I used to cough up clear or white phlegm in the mornings, but one day last week, I had a coughing attack where I brought up about a teaspoon or so of blood. It was bright red and scared me and my spouse. It has occurred a couple times in the morning since then, but not so much during the day. |
| Amount |  |
| Aggravated by what | Nothing in particular |
| Relieved by what | Nothing in particular, but it does seem to occur less as the day goes on. |
| Associated with what | Over the past few months, I seem more tired than I used to. I don’t have any specific weakness, I just feel like I can’t do as much as I used to. I’ve also lost some weight over the past couple months, probably about 15 pounds, but I haven’t been trying to lose weight. |
| Attitude (what does the patient think is the problem, and how does he/she feel about it) | I don’t know why the cough is worse or why I’m coughing up some blood now, but I’m worried that it could be cancer or something else that’s serious. |
| Overall course |  |
| REVIEW OF SYSTEMS: Significant positives and negatives | |
| Fever, chills, night sweats | No fevers or chills, but I had had some night sweats (i.e., soaked the sheets with sweat) a few times a week over the past month. |
| Weight loss | About 15 pounds in the last 4 months or so |
| Fatigue | I noticed that I get tired easier than I used to over the past few months (the past 3 to 4 months, if pressed on this), but I am still able to go to work and go about my day. |
| Loss of Appetite | My appetite has declined a bit in the past few months, but I still eat all meals as usual. |
|  | |
| Past medical history | - High blood pressure  - Chronic Obstructive Pulmonary Disease (COPD) |
| Medication allergies (Name and reaction) | None |
| Environmental allergies (Name and reaction) | None |
| Illnesses | None |
| Vaccinations | I’ve had all my regular vaccines, as far as I know. |
| Surgeries | None |
| Accidents/ injuries/ trauma | None |
| Hospitalization | I was hospitalized for an exacerbation of my COPD about 4 months ago, after I had the flu, which caused the exacerbation. |
|  | |
| Inclusive sexual and reproductive history | |
| Sexual practices  Sexual partners  Protection: Use of safer sex practices  Use of birth control if appropriate  Risk of intimate partner violence | Heterosexual  One current sexual partner (my spouse); two total lifetime partners  I do not use barrier protection. |
| Ob/GYN HISTORY (if SP is female): | Age of onset of menses: 14 years old  Age of menopause: 49 years old  Number of pregnancies: 2  Number of live births: 2  Number of miscarriages: 0  Number of abortions: 0 |
| Medications | - Hydrochlorothiazide (HCTZ) 12.5 milligrams daily, for high blood pressure  - Tiotropium 18 microgram capsule inhaled daily using meter dose inhaler, for COPD  - Albuterol MDI 90 micrograms per puff, used as needed every 4 - 6 hours, for shortness of breath or wheezing  - Fluticasone propionate 250 micrograms/Salmeterol 50 micrograms) 1 puff twice daily for COPD *(note: this medication was added after the recent hospitalization, and it will not be on the medication list that the student sees in the chart)* |
| Immunizations | X Tetanus  X Flu   - Hepatitis   X Pneumovax   - HPV - Other |
| Tobacco products:  X Cigarettes   - Cigar - Pipe - Chew - E-cigarettes | - Never - Past- year started/year quit   X Current   - Quantity: 1.5 packs per day - # of years: 35 years |
| Alcohol   - Beer - Wine - Liquor - Other | X Never   - Past- year started/year quit - Current   - Quantity   - # of years |
| Drugs   - Weed - Cocaine - Heroin - Meth - Other - IV - Inhalants - Other | X Never   - Past- year started/year quit - Current   - Quantity   - # of years |
| Diet (describe) | I try to eat healthy by eating a mix of protein and vegetables with few starches. |
| Exercise (describe) | I do not exercise routinely. |
| List any other important social history or information important to this case |  |
| Family history |  |
| Mother, Father, Siblings, Grandparents, and other significant findings. | - Mother: Living and is overweight with diabetes. She is otherwise healthy and is retired.  - Father: Living and is a smoker. He has advanced COPD and is frequently hospitalized for respiratory issues. He had a heart attack at age 53 but no other health conditions.  - Children: No known medical problems.  - Maternal Grandfather: He was a long-time smoker. He died at 72 after a long battle with lung cancer.  - Maternal Grandmother: Died from a heart attack at age 69.  - Paternal Grandmother: Deceased. Had dementia and died from pneumonia.  - Paternal Grandfather: Deceased. Unknown cause of death. |
|  |  |
| Physical Exam-  Vitals: Temp 99.6 F, BP 136/84, HR 80, RR 14, Pulse ox 92% on room air  General appearance: Sitting in the chair, occasional cough during encounter. Otherwise, appears comfortable.  Students will perform a targeted physical exam of your chest and lungs.  - If the student touches your neck and upper chest, just above the collarbone, in order to inspect them, give them the NECK card.  - If the student asks you to take deep breaths as they use their stethoscope to listen to your lungs, give them the LISTEN card.  - If the student taps on your chest, give them the TAP card.  - If the student places their hands on your chest and ask you to say something such as, “blue balloon” or “99,” give them the SPEAK card.  Aside from the lymph node and lung exam, the remaining physical examination findings will be normal. | |
|  |  |
| DIAGNOSIS AND DIFFERENTIAL |  |
| 1) Diagnosis with support from positive and negative history and PE findings | This patient has a new diagnosis of lung cancer. Lung cancer is most likely in this case due to the patient’s demographics, smoking history, slowly progressive cough with bloody sputum, and physical exam findings including the lymphadenopathy and presence of a pleural effusion. |
| 2) Differential with support from positive and negative history and PE findings | Other diagnoses to consider include infection, such as Tuberculosis, bacterial and fungal pneumonia, and rheumatologic diseases. |
| 3) |  |
| 4) MANAGEMENT OR DIAGNOSTIC PLAN | Appropriate evaluation in this case would include ordering a complete blood count, complete metabolic panel, and CT of the chest with contrast. |
| 5) |  |
| PROFESSIONALISM ISSUES OR CHALLENGES: | None |
|  |  |
|  |  |
|  |  |
|  |  |
|  |  |
|  |  |
|  |  |

| NECK  NECK |  | LISTEN  LISTEN |
| --- | --- | --- |
| *2cm fixed, non-mobile, non-tender right supraclavicular lymph node* |  | *Decreased breath sounds 1/3 of the way up on the right* |

| SPEAK  SPEAK |  | TAP  TAP |
| --- | --- | --- |
| *Decreased tactile fremitus 1/3 of the way up on the right* |  | *Dullness to percussion 1/3 of the way up on the right* |

**Case 2: Upper Airway Cough Syndrome and Asthma**

Patient Name: Pat Lopez

Chief Complaint: “shortness of breath”

Most likely Diagnosis and Differential with rationale from history and/or physical exam: This patient has upper airway cough syndrome as well as worsening of his/her baseline asthma. Upper airway cough syndrome is the most likely cause of the patient’s cough due to the history of allergies and similar symptoms in the past, as well as the physical exam findings including the bluish hue of the nasal turbinates, conjunctival injection, and oropharyngeal cobblestoning. This patient also has worsening of his/her asthma, as evidenced by the symptoms of worsening shortness of breath with minimal exertion and the diffuse expiratory wheezing heard on lung exam. Other diagnoses to consider include acute viral or bacterial infections including upper respiratory tract infection and pneumonia.

| SETTING: outpatient, in patient, ED, home, nursing home, rehab, group etc. | Primary care clinic |
| --- | --- |
| PATIENT PROFILE: | |
| Age range | 20-40 years |
| Religious/spiritual background | Any |
| Sex (e.g., male, female, intersex, transwoman, transman) | Any |
| Sexual Orientation (e.g., heterosexual, lesbian, gay, bisexual, pansexual, queer, asexual) | Any |
| Gender expression (e.g., man, woman, gender queer) | Any |
| Race/ethnicity: | Any |
| Physical description (e.g., BMI, height range) | Average BMI |
| Physical limitations | None |
| Patient appearance (e.g., disheveled, hospital gown, business casual, casual) | Hospital gown |
| Moulage + location (e.g., none, bruises, scars, body piercing, tattoos) | None |
| Affect (e.g., pleasant, cooperative) | Pleasant, cooperative |
| Family group (e.g., who is family, who they live with) | I live alone in an apartment. My parents live nearby. |
| Education | I completed high school. |
| Level of health literacy | Low |
| Employment, if any - present and past, noting any current stresses | I work as a cashier at a local restaurant. |
| Home/homeless - type of dwelling, number of stories, owned or rented | I live in a one-bedroom apartment that I rent. |
| Financial situation- any current stresses | No current stresses |
| Insurance Status (e.g., un/under/insured, public/private, HMO/PPO) | I am currently uninsured and get my medications through the local free pharmacy. |
| Habits (i.e., diet, exercise, caffeine, smoking, alcohol, drugs) | Diet: I eat a lot of low cost, easy to prepare foods, including frozen meals, cereal, and sandwiches.  Exercise: I usually run 2-3 miles a day, but I can’t run right now because of the shortness of breath.  Tobacco: I started smoking cigarettes at age 17 and have smoked one half pack per day since then.  Alcohol: None  Drugs: None |
| Activities (i.e., hobbies, sports, clubs, friends) | I enjoy spending time with my friends watching movies and playing games or visiting with my family. |
| Typical day - what is the usual daily routine | I typically get up around 8 am and go for a run, then get ready for work. Lately, I have not been running due to my symptoms. I typically work from around 11 am until after dinner, sometime around 8 pm. |

| CASE INFORMATION | |
| --- | --- |
| Chief Concern: What the patient will say when greeted by the student. The patient’s primary reason for seeking medical care often stated in his/own words. | I’ve been feeling short of breath. |
| Additional Concerns: Other, if any, concerns the patient has today (i.e., symptoms, requests, expectations, etc.) that will become part of set agenda. | Cough and runny nose/itchy eyes |
|  | |
| THE PATIENT STORY: The SP will be asked to tell their symptom story and the personal and emotion impact for each of their concerns. You will want to write this is the patient voice. The symptom story should be able to answer this question: “Tell me more about [chief concern/additional concern], starting at the beginning and bringing me up to now.”  The personal context should be able to answer questions concerning the broader personal/psychosocial context of symptoms, especially the patient beliefs/attributions.  The emotional context should be able to ask how are you doing with this, how does this make you feel, how has this affected you emotionally? IMPACT: How has this affected your life? How has this been for your family? | I’ve had this shortness of break for 2 or 3 weeks now. I’ve been using my albuterol inhaler more during the day and night, and I don’t think it’s really helping anymore. At first, the shortness of breath was worse when I was walking outside to my care, but it went away pretty quickly when I came inside or used my inhaler. Over the last week it has been much worse, where even a small amount of activity – inside or outside – brings on the shortness of breath.  Personal/emotional context: I have had similar symptoms in the past when my asthma was out of control, so I wonder if this is the problem.  Impact: The shortness of breath seems to come on more when I do any kind of activity (e.g., walking). Lately, I have not been running due to my symptoms. |
| HISTORY OF PRESENT ILLNESS: Although some of the HPI will be given in the patient’s symptom story, the learners will expand the story during the direct question section. Below describe the detailed history, usually about the chief concern, which the student must develop in order to make a useful assessment of the problem: | |
|  | |
| Onset (when; gradual or sudden) | The shortness of breath started 2 to 3 weeks ago and has been gradually getting worse. |
| Setting (what was going on or where was patient when symptoms first noticed?) | I wasn’t doing anything out of the ordinary when it started. |
| Duration (how long) |  |
| Time relationships (frequency, constant or intermittent) | The shortness of breath is intermittent; seems to come on more when I do any kind of activity. |
| Location | N/A |
| Radiation | N/A |
| Quality | N/A |
| Amount | N/A |
| Aggravated by what | Walking; seems to be worse when walking outside |
| Relieved by what | Going back inside and using the albuterol inhaler |
| Associated with what | - Cough: For the past week, I have been waking up at night coughing. The cough was dry at first, but now I am coughing up some clear, whitish colored phlegm.  - Runny nose and itchy eyes: The itchy eyes and runny nose started about a week prior to the shortness of breath. These are my typical allergy symptoms, and they typically come on around this time of year, but they seem to be worse this year. I used to take Flonase, but this year I’ve been trying Zyrtec. |
| Attitude (what does the patient think is the problem, and how does he/she feel about it) | I have had similar symptoms in the past when my asthma was out of control, so I wonder if this is the problem. |
| Overall course | The symptoms have been gradually worsening overall. |
| REVIEW OF SYSTEMS: Significant positives and negatives | |
| Fever, chills | None |
| Fatigue | I have had some mild fatigue over the past 2 to 3 weeks. |
| Nasal congestion/drainage and itchy eyes | Present for the past 3 to 4 weeks with white nasal discharge (as above) |
| Cough | Present for the past week with clear, white phlegm, worse at night (as above) |
|  | |
| Past medical history | - Asthma: diagnosed as a child; I have never been intubated for this (if asked).  - Seasonal allergies |
| Medication allergies (Name and reaction) | Penicillin – causes a severe rash with associated throat itching *(Note: there are no allergies listed in your chart; you will need to correct the student on this point at the appropriate time in the interview.)* |
| Environmental allergies (Name and reaction) | Hay/grass – runny nose, itchy eyes |
| Illnesses | None |
| Vaccinations | I believe I am up to date on all the usual vaccinations. |
| Surgeries | None |
| Accidents/ injuries/ trauma | None |
| Hospitalization | I have been hospitalized 3 times for asthma exacerbations, with my last hospitalization 2 years ago. |
|  | |
| Inclusive sexual and reproductive history | |
| Sexual practices  Sexual partners  Protection: Use of safer sex practices  Use of birth control if appropriate  Risk of intimate partner violence | Heterosexual  No current sexual partners (two total lifetime partners)  I use barrier protection (condoms) |
| Ob/GYN HISTORY (if SP female): | Age of onset of menses: 15  Age of menopause: N/A  Number of pregnancies: 0  Number of live births: 0  Number of miscarriages: 0  Number of abortions: 0 |
| Medications | - Fluticasone propionate 100 micrograms/Salmeterol 50 micrograms) 1 inhalation twice daily, for asthma  - Albuterol MDI 90 micrograms; used as needed every 4 to 6 hours, for shortness of breath or wheezing  - Fluticasone Nasal Spray 55 micrograms 1 spray per nostril once daily (*if asked:* I am not taking this currently, as I ran out; instead, I am taking Cetirizine (Zyrtec) 10 milligrams daily, which I bought over the counter at my local pharmacy) |
| Immunizations | X Tetanus  X Flu  X Hepatitis   - Pneumovax - HPV - Other |
| Tobacco products:  X Cigarettes   - Cigar - Pipe - Chew - E-cigarettes | - Never - Past- year started/year quit   X Current   - Quantity: one half pack per day - # of years: depends on the age of the patient; started smoking at age 17 |
| Alcohol   - Beer - Wine - Liquor - Other | X Never   - Past- year started/year quit - Current   - Quantity   - # of years |
| Drugs   - Weed - Cocaine - Heroin - Meth - Other - IV - Inhalants - Other | X Never   - Past- year started/year quit - Current   - Quantity   - # of years |
| Diet (describe) | I eat a lot of low cost, easy to prepare foods, including frozen meals, cereal, and sandwiches. |
| Exercise (describe) | I usually run 2 to 3 miles a day, but I can’t run right now because of the shortness of breath. |
| List any other important social history or information important to this case |  |
| Family history |  |
| Mother, Father, Siblings, Grandparents, and other significant findings. | - Mother: Living and is a smoker. She is in remission from left sided breast cancer. Currently treated for high blood pressure, but no other conditions.  - Father: Living. He has asthma and eczema.  - Maternal Grandfather: He was a long-time smoker is treated for emphysema. He had a stroke 5 years ago.  - Maternal Grandmother: Died from a heart attack at age 74.  - Paternal Grandmother: Deceased. Unknown cause of death.  - Paternal Grandfather: Deceased after a car accident in his 40s. |
|  |  |
| Physical Exam-  Vitals: Temp 98.4 F, BP 125/72, HR 95, RR 18, Pulse ox 96% on room air  General appearance: comfortable appearing  Students will perform a targeted physical exam of your head, eyes, ears, nose, chest, and lungs.   - If they look in your eyes with a light, give them the EYES card. - If they look in your nose, give them the NOSE card. - If they look in your throat with a light, give them the THROAT card. - If they listen to your lungs, give them the LISTEN card.   All other physical exam findings will be normal. | |
|  |  |
| DIAGNOSIS AND DIFFERENTIAL |  |
| 6) Diagnosis with support from positive and negative history and PE findings | This patient has upper airway cough syndrome as well as worsening of his/her baseline asthma. Upper airway cough syndrome is the most likely cause of the patient’s cough due to the history of allergies and similar symptoms in the past, as well as the physical exam findings including the bluish hue of the nasal turbinates, conjunctival injection, and oropharyngeal cobblestoning. This patient also has worsening of his/her asthma, as evidenced by the symptoms of worsening shortness of breath with minimal exertion and the diffuse expiratory wheezing heard on lung exam. |
| 7) Differential with support from positive and negative history and PE findings | Other diagnoses to consider include acute viral or bacterial infections including upper respiratory tract infection and pneumonia. |
| 8) |  |
| 9) MANAGEMENT OR DIAGNOSTIC PLAN | Appropriate management in this case would include restarting the patient’s Flonase, continuing the cetirizine, and consideration of steroids for treatment of an asthma exacerbation. This patient likely needs an increase in the daily dose of Advair as well as pulmonary function tests, if not completed within the past year. |
| 10) |  |
| PROFESSIONALISM ISSUES OR CHALLENGES: | None |
|  |  |
|  |  |
|  |  |
|  |  |
|  |  |
|  |  |
|  |  |

| NOSE  NOSE |  | EYES  EYES |
| --- | --- | --- |
| *Bluish hue to the nasal turbinates bilaterally with clear drainage* |  | *Conjunctival injection bilaterally with infraorbital edema* |

| LISTEN  LISTEN |  | THROAT  THROAT |
| --- | --- | --- |
| *Diffuse expiratory wheezing with prolonged expiratory phase of the respiratory cycle* |  | *Posterior oropharyngeal cobblestoning without tonsillar erythema or exudates* |

**Case 3: Pneumonia**

Patient Name: Riley Yates

Chief Complaint: “cough”

Most likely Diagnosis and Differential with rationale from history and/or physical exam: This patient has acute bacterial pneumonia. This diagnosis is supported by the history of acute onset fever and cough with productive sputum, as well as the physical examination findings of a right sided consolidation of the lung. Other diagnoses to consider include viral or fungal etiologies of pneumonia, upper respiratory tract infection, or sinusitis.

| SETTING: outpatient, in patient, ED, home, nursing home, rehab, group etc. | Primary care clinic |
| --- | --- |
| PATIENT PROFILE: | |
| Age range | 40-60 years |
| Religious/spiritual background | Any |
| Sex (e.g., male, female, intersex, transwoman, transman) | Any |
| Sexual Orientation (e.g., heterosexual, lesbian, gay, bisexual, pansexual, queer, asexual) | Any |
| Gender expression (e.g., man, woman, gender queer) | Any |
| Race/ethnicity: | Any |
| Physical description (e.g., BMI, height range) | Average BMI |
| Physical limitations | None |
| Patient appearance (e.g., disheveled, hospital gown, business casual, casual) | Hospital gown |
| Moulage + location (e.g., none, bruises, scars, body piercing, tattoos) | None |
| Affect (e.g., pleasant, cooperative) | Pleasant, cooperative |
| Family group (e.g., who is family, who they live with) | I live with my spouse, and I am now taking care of my mother who has moved into our home. |
| Education | College degree in business |
| Level of health literacy | Average |
| Employment, if any - present and past, noting any current stresses | I work as a mortgage broker for a local bank. |
| Home/homeless - type of dwelling, number of stories, owned or rented | I live in a single-family home, which we own. |
| Financial situation- any current stresses | I am currently under significant stress due to caring for my mother, who is on hospice for metastatic breast cancer. |
| Insurance Status (e.g., un/under/insured, public/private, HMO/PPO) | I have insurance through my employer. |
| Habits (i.e., diet, exercise, caffeine, smoking, alcohol, drugs) | Diet: I eat a lot of easy to prepare foods, including frozen meals, sandwiches, and fast food.  Exercise: I used to ride my bike 3 times a week, but have been unable to do so lately due to stress at home.  Tobacco: None (never smoker)  Alcohol: I never used to drink alcohol, but started drinking over the past few months due to the stress of working and caring for my mother with cancer. I am now drinking 3 to 4 glasses of wine per night, and am trying to cut back, as I realize I am drinking too much.  Drugs: None |
| Activities (i.e., hobbies, sports, clubs, friends) | I enjoy swimming in the summertime and riding my bike throughout the year when the weather allows. |
| Typical day - what is the usual daily routine | On a usual day, I wake up around 6 am, get ready for work and check on my mother. I typically work from 9 am to 5 pm, and come home to have dinner with my spouse and spend time with my mother. |

| CASE INFORMATION | |
| --- | --- |
| Chief Concern: What the patient will say when greeted by the student. The patient’s primary reason for seeking medical care often stated in his/own words. | I’ve had this cough that just keeps getting worse. |
| Additional Concerns: Other, if any, concerns the patient has today (i.e., symptoms, requests, expectations, etc.) that will become part of set agenda. | Fever, fatigue |
|  | |
| THE PATIENT STORY: The SP will be asked to tell their symptom story and the personal and emotion impact for each of their concerns. You will want to write this is the patient voice. The symptom story should be able to answer this question: “Tell me more about [chief concern/additional concern], starting at the beginning and bringing me up to now.”  The personal context should be able to answer questions concerning the broader personal/psychosocial context of symptoms, especially the patient beliefs/attributions.  The emotional context should be able to ask how are you doing with this, how does this make you feel, how has this affected you emotionally? IMPACT: How has this affected your life? How has this been for your family? | I’ve had this cough for 4 days now, and it’s keeping me up at night. Over the past few days, it has continued to get worse, and I’m coughing up some thick, green phlegm.  Personal/emotional context: I felt so bad yesterday that I called out of work, which is really unusual for me. I don’t feel up to doing much more than watching TV and resting on the couch. I am concerned that I have an infection and think that I need an antibiotic.  Impact: Overall, I feel tired and unwell. It’s hard to get up and go about my day due to the symptoms. |
| HISTORY OF PRESENT ILLNESS: Although some of the HPI will be given in the patient’s symptom story, the learners will expand the story during the direct question section. Below describe the detailed history, usually about the chief concern, which the student must develop in order to make a useful assessment of the problem: | |
|  | |
| Onset (when; gradual or sudden) | 4 days ago; the cough started slowly and has been gradually getting worse |
| Setting (what was going on or where was patient when symptoms first noticed?) | I didn’t do anything out of the ordinary when it started, and I don’t recall being around anyone who was sick. |
| Duration (how long) | 4 days |
| Time relationships (frequency, constant or intermittent) | The cough is intermittent throughout the day. |
| Location | N/A |
| Radiation | N/A |
| Quality | Initially the cough was dry, but then I started coughing up some phlegm, which was yellow at first. Now it looks thick and green. *If asked:* I have not seen any blood in it. |
| Amount | N/A |
| Aggravated by what | Activity, deep breathing, talking too much |
| Relieved by what | Nothing has helped the cough. I tried some cough syrup that I bought at the drug store, but it didn’t seem to help. |
| Associated with what | - Fever: I’ve felt hot and sweaty a few times each day since the cough started. I don’t have a thermometer at home, so I haven’t checked my temperature, but I’m pretty sure I have had a fever.  - Fatigue: Overall, I feel tired and unwell. It’s hard to get up and go about my day due to the symptoms. |
| Attitude (what does the patient think is the problem, and how does he/she feel about it) | I am concerned that I have an infection and think that I need an antibiotic. |
| Overall course | Overall, the symptoms have continued to worsen over the past 4 days since they began. |
| REVIEW OF SYSTEMS: Significant positives and negatives | |
| Fever | I have felt feverish since the cough started 4 days ago, but have not checked my temperature (as above). |
| Fatigue | Present for the past week; I haven’t been sleeping well and think this is all due to caring for my mother. |
| Nausea/vomiting | I vomited once or twice in the past week. This occurred after drinking an extra glass of wine and has prompted me to cut back. |
|  | |
| Past medical history | - Myasthenia Gravis: diagnosed 2 years ago, currently well controlled  - Diabetes type 2: diagnosed 5 years ago, well controlled |
| Medication allergies (Name and reaction) | None |
| Environmental allergies (Name and reaction) | None |
| Illnesses | None |
| Vaccinations | I believe I am up to date on my vaccinations. |
| Surgeries | None |
| Accidents/ injuries/ trauma | None |
| Hospitalization | I was hospitalized 2 years ago for weakness, at which time I was diagnosed with myasthenia gravis. |
|  | |
| Inclusive sexual and reproductive history | |
| Sexual practices  Sexual partners  Protection: Use of safer sex practices  Use of birth control if appropriate  Risk of intimate partner violence | Heterosexual  One current sexual partner (spouse); 3 total lifetime partners.  I do not use barrier protection. |
| Ob/GYN HISTORY (if SP female): | Age of onset of menses: 13 years  Age of menopause: 50 years (if applicable)  Number of pregnancies: 0  Number of live births: 0  Number of miscarriages: 0  Number of abortions: 0 |
| Medications | - Pyridostigmine 120 milligrams three times a day, for Myasthenia Gravis  - Metformin 1 gram twice a day, for diabetes  - Guaifenesin 400 milligrams as needed for cough (I took 2 or 3 times, with no effect) |
| Immunizations | X Tetanus  X Flu  X Hepatitis   - Pneumovax - HPV - Other |
| Tobacco products:   - Cigarettes - Cigar - Pipe - Chew - E-cigarettes | X Never   - Past- year started/year quit - Current   - Quantity   - # of years |
| Alcohol   - Beer - Wine - Liquor - Other | - Never - Past- year started/year quit   X Current   - Quantity: 3-4 glasses of wine per night - # of years: 3-4 months |
| Drugs   - Weed - Cocaine - Heroin - Meth - Other - IV - Inhalants - Other | X Never   - Past- year started/year quit - Current   - Quantity   - # of years |
| Diet (describe) | I eat a lot of easy to prepare foods, including frozen meals, sandwiches, and fast food. |
| Exercise (describe) | I used to ride my bike 3 times a week, but have been unable to do so lately due to stress at home. |
| List any other important social history or information important to this case |  |
| Family history |  |
| Mother, Father, Siblings, Grandparents, and other significant findings. | - Mother: Living and is a smoker. She was diagnosed with metastatic breast cancer 2 months ago, and I’ve been taking care of her in my home. She is now on hospice and likely to pass away in the upcoming weeks.  - Father: deceased from a heart attack.  - Maternal Grandfather: unknown; died at a young age during military service  - Maternal Grandmother: Died from breast cancer at age 74.  - Paternal Grandmother: Deceased. Unknown cause of death.  - Paternal Grandfather: Deceased after a stroke in his 60s. |
|  |  |
| Physical Exam-  Vitals: Temp 100.7 F, BP 115/78, HR 92, RR 12, Pulse ox 93% on room air  General appearance: you appear mildly uncomfortable, with occasional cough throughout the interview  Students will perform a targeted physical exam of your chest and lungs.   - If they listen to your lungs, give them the LISTEN card. - If they tap on your lungs, give them the TAP card. - If they ask you to say blue balloon or 99 or other phrase while placing their hands on your back, give them the SPEAK card.   All other physical exam findings will be normal. | |
|  |  |
| DIAGNOSIS AND DIFFERENTIAL |  |
| Diagnosis with support from positive and negative history and PE findings | This patient has acute, bacterial pneumonia. This diagnosis is supported by the history of acute onset fever and cough with productive sputum, as well as the physical examination findings of a right-sided consolidation of the lung. |
| Differential with support from positive and negative history and PE findings | Other diagnoses to consider include viral or fungal etiologies of pneumonia, upper respiratory tract infection, or sinusitis. |
|  |  |
| MANAGEMENT OR DIAGNOSTIC PLAN | Appropriate management in this case would include ordering a chest x-ray, complete blood count, as well as empiric treatment with oral antibiotics. Of note, this patient should not be started on a floroquinolone due to his history of Myasthenia Gravis. |
|  |  |
| PROFESSIONALISM ISSUES OR CHALLENGES: | None |
|  |  |

| LISTEN  LISTEN |  | TAP  TAP |
| --- | --- | --- |
| *Decreased breath sounds at the*  *right base, 1/3 of the way up* |  | *Dullness to percussion 1/3 of*  *the way up on the right* |

SPEAK

SPEAK

*Increased tactile fremitus 1/3 of*

*the way up on the right*

**Case 4: Chronic Obstructive Pulmonary Disease**

Patient Name: Peyton Tran

Chief Complaint: “shortness of breath”

Most likely Diagnosis and Differential with rationale from history and/or physical exam: This patient has undiagnosed Chronic Obstructive Pulmonary Disease (COPD). This is the most likely diagnosis based on the patient’s significant smoking history, shortness of breath with morning cough, and physical exam findings of wheezing with prolonged expiratory phase and mild hypoxia. Other diagnoses to consider include asthma, atypical pneumonia, and coronary artery disease.

| SETTING: outpatient, in patient, ED, home, nursing home, rehab, group etc. | Primary care clinic |
| --- | --- |
| PATIENT PROFILE: | |
| Age range | 50-70 years |
| Religious/spiritual background | Any |
| Sex (e.g., male, female, intersex, transwoman, transman) | Any |
| Sexual Orientation (e.g., heterosexual, lesbian, gay, bisexual, pansexual, queer, asexual) | Any |
| Gender expression (e.g., man, woman, gender queer) | Any |
| Race/ethnicity: | Any |
| Physical description (e.g., BMI, height range) | Average BMI |
| Physical limitations | None |
| Patient appearance (e.g., disheveled, hospital gown, business casual, casual) | Hospital gown |
| Moulage + location (e.g., none, bruises, scars, body piercing, tattoos) | None |
| Affect (e.g., pleasant, cooperative) | Pleasant, cooperative |
| Family group (e.g., who is family, who they live with) | I live alone. |
| Education | Graduated high school |
| Level of health literacy | Low |
| Employment, if any - present and past, noting any current stresses | Driver for a ride-sharing service (e.g., Uber) |
| Home/homeless - type of dwelling, number of stories, owned or rented | I live in an apartment that I rent. |
| Financial situation- any current stresses | No current stresses |
| Insurance Status (e.g., un/under/insured, public/private, HMO/PPO) | I am uninsured. |
| Habits (i.e., diet, exercise, caffeine, smoking, alcohol, drugs) | Diet: I eat a lot of easy to prepare foods, including frozen meals, sandwiches, and fast food.  Exercise: I do not exercise routinely aside from walking the dog a couple times a day.  Tobacco: I started smoking at age 15 and have smoked 1-2 packs per day since then. *If asked:* I am trying to quit, but it’s been very difficult, and I don’t know how to stop smoking.  Alcohol: None  Drugs: None |
| Activities (i.e., hobbies, sports, clubs, friends) | I enjoy spending time with my dog on walks and playing at the local park. I also enjoy watching movies with friends. |
| Typical day - what is the usual daily routine | On a typical day, I sleep in until around 10am or so, then watch some television and do any work around the apartment that needs to be done. I start driving in the midafternoon, and continue working until late at night, depending on how busy it is. |

| CASE INFORMATION | |
| --- | --- |
| Chief Concern: What the patient will say when greeted by the student. The patient’s primary reason for seeking medical care often stated in his/own words. | I feel short of breath when I walk. |
| Additional Concerns: Other, if any, concerns the patient has today (i.e., symptoms, requests, expectations, etc.) that will become part of set agenda. | I want to quit smoking, and I need help with this. |
|  | |
| THE PATIENT STORY: The SP will be asked to tell their symptom story and the personal and emotion impact for each of their concerns. You will want to write this is the patient voice. The symptom story should be able to answer this question: “Tell me more about [chief concern/additional concern], starting at the beginning and bringing me up to now.”  The personal context should be able to answer questions concerning the broader personal/psychosocial context of symptoms, especially the patient beliefs/attributions.  The emotional context should be able to ask how are you doing with this, how does this make you feel, how has this affected you emotionally? IMPACT: How has this affected your life? How has this been for your family? | About a month ago, I started feeling short of breath. I used to be able to take my dog on walks around the neighborhood without any problem, but now I can only walk one block before I have to stop and rest to catch my breath. It seems to be getting worse day by day.  Personal and emotional contexts: I have never had shortness of breath like this before, and I don’t like feeling limited in what I can do outside of my home. I don’t know what is causing the shortness of breath, but I am starting to wonder if it could be related to my smoking. I know that I should have stopped smoking a long time ago, and I’m afraid that I am starting to have serious lung damage because of it.  Impact: It comes and goes with activity. I feel short of breath when walking to the mailbox or walking my dog. I can cook and do other daily activities without getting short of breath. |
| HISTORY OF PRESENT ILLNESS: Although some of the HPI will be given in the patient’s symptom story, the learners will expand the story during the direct question section. Below describe the detailed history, usually about the chief concern, which the student must develop in order to make a useful assessment of the problem: | |
|  | |
| Onset (when; gradual or sudden) | One month ago, gradual |
| Setting (what was going on or where was patient when symptoms first noticed?) | I wasn’t doing anything unusual when the symptom started. |
| Duration (how long) | One month |
| Time relationships (frequency, constant or intermittent) | It comes and goes with activity. I feel short of breath when walking to the mailbox or walking my dog. I can cook and do other daily activities without getting short of breath. |
| Location | N/A |
| Radiation | N/A |
| Quality | It feels like I can’t get enough air. |
| Amount | N/A |
| Aggravated by what | Exercise |
| Relieved by what | Rest |
| Associated with what | I’ve had a mild cough when I wake up in the morning. I sometimes cough up a little clear phlegm in the morning, but then nothing else throughout the day. |
| Attitude (what does the patient think is the problem, and how does he/she feel about it) | I have never had shortness of breath like this before, and I don’t like feeling limited in what I can do outside of my home. I don’t know what is causing the shortness of breath, but I am starting to wonder if it could be related to my smoking. I know that I should have stopped smoking a long time ago, and I’m afraid that I am starting to have serious lung damage because of it. |
| Overall course | Since I first noticed the shortness of breath one month ago, it has slowly been getting worse. |
| REVIEW OF SYSTEMS: Significant positives and negatives | |
| Fevers, chills | None |
| Weight changes | I’ve lost 2-3 pounds over the last month or two, even though I have not been trying to lose weight. |
| Decreased appetite | I’ve noticed this for the past 2-3 months. |
| Chest pain | None |
|  | |
| Past medical history | - High blood pressure: diagnosed 5 years ago  - Gout – I had a mild flare in my left big toe recently that I treated myself with ibuprofen  - Arthritis of the knees: diagnosed 5 years ago; controlled with ibuprofen |
| Medication allergies (Name and reaction) | None |
| Environmental allergies (Name and reaction) | None |
| Illnesses | None |
| Vaccinations | I am up to date on my vaccines, as far as I know. |
| Surgeries | None |
| Accidents/ injuries/ trauma | None |
| Hospitalization | None |
|  | |
| Inclusive sexual and reproductive history | |
| Sexual practices  Sexual partners  Protection: Use of safer sex practices  Use of birth control if appropriate  Risk of intimate partner violence | Heterosexual  No current sexual partners (5 total lifetime partners)  I use barrier protection when I am sexually active. |
| Ob/GYN HISTORY (if SP is female): | Age of onset of menses 13  Age of menopause 52 (if applicable)  Number of pregnancies 0  Number of live births 0  Number of miscarriages 0  Number of abortions 0 |
| Medications | - Amlodipine 10 milligrams daily for high blood pressure; *if asked:* I ran out of this a couple weeks ago and need a refill today.  - Allopurinol 100 milligrams daily, for gout  - Ibuprofen 400 milligrams, three times a day as needed, for arthritis; I recently took twice this amount when my gout was acting up, but haven’t taken any for the past week. |
| Immunizations | X Tetanus  X Flu   - Hepatitis - Pneumovax - HPV - Other |
| Tobacco products:  X Cigarettes   - Cigar - Pipe - Chew - E-cigarettes | - Never - Past- year started/year quit   X Current   - Quantity: 1-2 packs per day - # of years: since age 15 |
| Alcohol   - Beer - Wine - Liquor - Other | X Never   - Past- year started/year quit - Current   - Quantity   - # of years |
| Drugs   - Weed - Cocaine - Heroin - Meth - Other - IV - Inhalants - Other | X Never   - Past- year started/year quit - Current   - Quantity - # of years |
| Diet (describe) | I eat a lot of easy to prepare foods, including frozen meals, sandwiches, and fast food. |
| Exercise (describe) | I do not exercise routinely aside from walking the dog a couple times a day. |
| List any other important social history or information important to this case |  |
| Family history |  |
| Mother, Father, Siblings, Grandparents, and other significant findings. | - Mother: deceased from a stroke in her 60s  - Father: Living. He has COPD and heart disease  - Maternal Grandfather: deceased from colon cancer  - Maternal Grandmother: deceased from complications of diabetes  - Paternal Grandmother: deceased at a young age from complications of childbirth  - Paternal Grandfather: deceased from prostate cancer |
|  |  |
| Physical Exam-  Vital signs: Temp 97.4 F, BP 145/88, HR 74, RR 14, Pulse ox 90% on room air  General appearance: comfortable, no distress  Students will perform a targeted physical exam of your chest and lungs.  - If they listen to your lungs, give them the LISTEN card.  - All other physical exam findings will be normal. | |
|  |  |
| DIAGNOSIS AND DIFFERENTIAL |  |
| Diagnosis with support from positive and negative history and PE findings | This patient has undiagnosed Chronic Obstructive Pulmonary Disease (COPD). This is the most likely diagnosis based on the patient’s significant smoking history, shortness of breath with morning cough, and physical exam findings of wheezing with prolonged expiratory phase and mild hypoxia. |
| Differential with support from positive and negative history and PE findings | Other diagnoses to consider include asthma, atypical pneumonia, and coronary artery disease. |
|  |  |
| MANAGEMENT OR DIAGNOSTIC PLAN | Appropriate management in this case would include ordering a chest x-ray and pulmonary function testing (PFTs). Initial treatment could include a trial of a short acting inhaled beta agonist, such as albuterol, while awaiting PFT results. It would also be reasonable to consider a short course of oral steroids. In addition, the patient should be counseled regarding smoking cessation and provided pharmacologic therapy (nicotine replacement, bupropion, or varenicline) if the patient agrees. |
|  |  |
| PROFESSIONALISM ISSUES OR CHALLENGES: | None |

LISTEN

LISTEN

*Diffuse expiratory wheezing with*

*prolonged expiratory phase of*

*the respiratory cycle*
