# Supplementary material for: Integrating the Electronic Health Record Into Patient Encounters: An Introductory Standardized Patient Exercise for Preclinical Medical Students
Source: MedEdPORTAL. 2022 Jan 5;18:11209. doi: 10.15766/mep_2374-8265.11209 (PMC8727442; doi:10.15766/mep_2374-8265.11209)
Supplement: Supplementary file 1 — Door Charts.docxSimulated EHR.pptxSession Logistics.docxSP Scripts and Instructions.docxPreclass Student Notes.docxPerspectives From Faculty on EHR.mp4Faculty Instructions.docxEHR Skills - Good Example.mp4EHR Skills - Bad Example.mp4Learner Evaluation.docx [file mep_2374-8265.11209-s001.zip › E. Preclass Student Notes.docx]

**The Electronic Health Record and the Doctor-Patient Relationship**

**Session overview**

| **Time** | **Activity** |
| --- | --- |
| First 15 minutes | Overview of the doctor-patient relationship & interacting with the EHR |
| The next 2 hours, 20 minutes | Simulated “urgent care” clinic: history taking, DPR, & PE practice; approach to “cough” and “dyspnea” |

**Learning Objectives:**

By the end of this activity, learners will be able to:

1. Practice communication strategies to effectively navigate the doctor-patient relationship while interacting with the electronic health record (EHR).

2. Verify information from a simulated EHR while obtaining history from a standardized patient during a simulated clinical encounter.

3. Perform additional, focused history taking based on data verified from the EHR.

**Pre-class materials**

**Introduction**

Simply put, the *electronic health record* (EHR) is a digital version of a patient’s paper chart. These digital records can typically be accessed in real time during patient care and in a variety of healthcare settings to maintain all a patient’s data in a single place for a given healthcare entity. Increasingly, EHRs have capabilities to facilitate certain patient care tasks rendered by physicians, nurses, and others. A few examples include the ability for the EHR to perform computerized physician order entry (CPOE), electronic medication prescribing directly to pharmacies in the community, and automation of the process for nurses to dispense to hospitalized patients. Lastly, EHRs are increasingly able to facilitate secure provider-to-provider and provider-to-patient messaging.

As the EHR increasingly has a presence in patient care, it has become a more visible part of the patient experience and inherently has impacted the modern-day doctor-patient relationship (DPR). Unfortunately, there is a growing body of medical literature that the increasingly common use of the EHR during a patient care encounter oftentimes diminishes the doctor-patient relationship and the effectiveness of communication between the clinician and the patient.

In Elizabeth Toll’s article, “A piece of my mind: The cost of technology” (JAMA 2012;307:2497-8), she shows an illustration drawn by a young child who accompanied his mother to a doctor’s visit and drew what was going on in the room during the encounter. It captured the essence of the unintended impact of the EHR on the DPR. This picture galvanized the national conversation amongst physicians on the impact the EHR is having on the DPR. The physician depicted in this drawing was a resident physician at Brown University at the time and was known by the faculty by his genuine sincerity, kindness, and humility. He is a young physician and not too far removed from current day medical students and residents alike. He has now completed his residency training. He has since served as a champion for ways to remain patient-focused during patient care encounters that increasingly necessitate the use of the EHR.

In this lesson, we will focus on strategies to maintain the doctor-patient relationship while introducing the reality of the EHR as a visible part of the patient encounter.

**DPR and the EHR: Strategies for Remaining Focused on the Patient**

You have already learned the fundamental concepts and strategies that you’ll need to remain attentive, empathic, and patient-centered. Skills such as agenda setting, identifying empathic opportunities, exploring the patients perspective of illness, and others you have practiced are strategies to remain patient-focused. You are already familiar with these fundamental skills, and adding in the EHR will only require just a bit more mindfulness made easier with a few “tips” and strategies mentioned below.

Below is an acronym adapted from the University of Chicago with strategies that can help the clinician to remain focused on the patient during patient encounters while working with the EHR:

**Honor the “Golden Minute”:** Make the start of the visit completely technology free. Greet the patient, start with their concerns, and establish an agenda for the visit before engaging technology.

**Use the “Triangle of Trust”:** Create a triangle configuration that puts you, the patient and the computer screen at each of the three corners. This allows you to look at both the patient and screen without shifting your body.

**Maximize patient interaction:** Encourage patient interaction. Pause for questions and clarification. Allow time for questions and to verify understanding.

**Acquaint yourself with chart:** Review the chart before you enter the room to prepare, inform, and contextualize your visit.

**Nix the screen:** When discussing sensitive information, completely disengage from the EHR (look at the patient, turn away from screen, take hands off keyboard, etc.).

**Let the patient look on:** Share things on the screen with your patients.

**Eye contact:** Maintain eye contact with patients as much as possible. Treat patient encounters as you would a conversation with friends or family members.

**Value the computer:** Praise the **benefits** of the EHR, and take advantage of opportunities to use technology as a tool to engage patients (e.g., pull up lab result to review together, utilize graphics, etc.).

**Explain what you’re doing:** Be transparent about everything you do. Avoid long silences, and aim for conversational EMR use by explaining what you are doing as you are doing it.

**Log off:** At the end of the visit, log off of the patient’s chart while they are still in the exam room. This reassures the patient that their medical information is secure.

*Source: Alkureishi MA LW, Farnan J, Arora V. Breaking away from the iPatient to care for the real patient: Implementing a patient-centered EMR use curriculum. MedEdPORTAL 2014:9953.*

**Perspectives from Experienced Faculty on the EHR:**

In addition to these notes, we have also developed a perspectives video with selected excerpts from experienced faculty at our institution on navigating the doctor-patient relationship while interacting with the EHR. While reviewing this video include elements of this strategy, we encourage you to remain mindful of the HUMAN LEVEL. Many of the strategies wrapped into HUMAN LEVEL were encouraged by our own faculty too.

Additionally, please also look for other themes and actionable suggestions made by faculty. In our creation of the videos, we noticed many materialize but we want to reinforce a couple that are particularly important:

1) **“Trust but verify”:** The EHR is a rich resource for data-gathering. Use of the EHR for in advance of a patient visit is commonplace and when appropriate can aid. However, this EHR data must be verified with the patient to ensure it is accurate and up to date.
2) **Ask Permission**: Before logging into the computer in the room, ask the patient for permission to do so since it will create a brief interruption early in the visit when you are typically engaging the patient in rapport building. Asking permission allows you to explain the purpose of using the EHR during the visit and explain to the patient the potential benefits for the encounter.

**Example Videos:**

Please watch videos “EHR Skills Video Good Example” and “EHR Skills Video Bad Example.” (Appendices H and I). We developed videos demonstrating helpful versus counter-productive techniques for interacting with patients and the EHR during encounters. Please consider all of the EHR specific strategies discussed earlier and look for whether or not these strategies were effectively implemented.

Note that there are a variety of styles that our faculty use to remain patient-focused during their patient care encounters. Remember, that “one size does not fit all” patients, physicians, settings of care, etc.

Consider the following questions while watching the example videos:

1) Which elements of HUMAN LEVEL did you see used in the good versus the bad example? Did use of these strategies appear to improve the provider-patient interaction?
2) Was the strategy of “trust but verify” present in the videos? Could you determine whether or not certain information discussed with the patient had been obtained antecedent to the visit?
3) Overall, what features distinguished the good from the bad performance? What key take-aways do you think come from these examples?

**In-class activities:**

**Part 1: Review of content from pre-class assigned materials**

**Please come prepared to discuss important points from “Electronic Medical Record and the Doctor-Patient Relationship” notes and reflections on the video, “**Perspectives from Experienced Faculty on the EHR.”

**Part 2: Simulated “Urgent Care Clinic” Exercise**

*NOTE: it is important that this activity begins promptly on time for all student groups, and that all groups stay on schedule as directed.*

The goal of this exercise is for you to assume the role of the physician in a simulated urgent care clinic and practice the strategies discussed in their pre-class assignments and preceding discussion. This exercise is designed for small groups of 1 faculty and up to 4 students.

**Logistical overview and timing:**

This activity will include a series of 4 patient encounters, so each student in your group should have an opportunity to lead a history and perform a PE with an SP at least once.  Each iteration of the activity will take place as follows:

- **SP encounter** (first 20 minutes): One student will lead each 20-minute encounter with an SP; meanwhile, the faculty and other students will observe the encounter and prepare feedback for the interviewer.  See below for a description of the expected tasks for students to perform during these encounters.
- **Debrief** (next 10 minutes): Following each encounter, there will be a 10-minute debrief period for feedback; involve the observing students in the discussion.
- **Transition time** (next 5 minutes): 5 minutes are allotted prior to the next encounter for the next student interviewer to proceed to their assigned exam room for the next encounter and review the EHR and door chart.

For each case, the interviewing student will proceed to the assigned exam room for the applicable case, according to your group’s assigned room rotation schedule.

It may be helpful to assign particular tasks to each observer, so that they can focus on one or two unique aspects of the encounter (e.g., positioning of individuals/computer screen in the room; nonverbal cues; verification of information found in the EHR; history taking approach for the HPI, etc.).

**SP encounter tasks:** Specific student tasks for these encounters include:

1. **Practice greeting and connecting with the patient.**
2. **Practice using open-ended questions to elicit the history of present illness and characterize the associated symptoms.**
3. **Practice reviewing and updating the past medical, family, and social histories, medications, and allergies utilizing the EHR:**

Note that, for these cases, students will not be in the role of the patient’s primary care physician; therefore, it will be important for them to verify the patient’s pertinent health history, medications, allergies and social history (for the latter, instruct students to focus on tobacco use and the patient’s occupation).

Instruct students to use the information provided in the simulated EHR on the in-room computer as a starting point, and to “trust but verify” the information they find in this EHR with the patient during the interview.

This exercise will work best if the EHR (which is a powerpoint file) is kept in SLIDESHOW mode, and the student simply clicks the applicable links within each slide to navigate within the EHR. If the student accidentally ends the “show,” instruct the student to put the file back into “slideshow” mode.

4. **Perform a physical exam, including vital signs and a targeted physical exam for the complaint.**

Students can determine, using their differential diagnosis for each case, which physical exam maneuvers seem most relevant to determining the source of the patient’s symptoms. For the cases in this set, this should at least include vital signs and a lung exam.

**Debrief**

Immediately following each patient encounter, student interviewers should return to your group’s assigned meeting room, to debrief with you and the other students in your small group. Start by prompting the student interviewer to reflect on the encounter; for example:

- *How did it go?*
- *What did you find most challenging?*
- *What might you do differently next time?*

Next, prompt the observing students in your group to provide feedback to the student interview, based on their observations of the encounter.

Finally, provide your own perspective of the student interviewer’s performance to fill in any gaps not yet mentioned by others in the group. Focus on 1 or 2 key aspects of the student’s performance, including encouragement for tasks s/he performed well. Connect the students’ observations to your own experiences in clinical practice, where applicable.
