# Supplementary material for: Integrating the Electronic Health Record Into Patient Encounters: An Introductory Standardized Patient Exercise for Preclinical Medical Students
Source: MedEdPORTAL. 2022 Jan 5;18:11209. doi: 10.15766/mep_2374-8265.11209 (PMC8727442; doi:10.15766/mep_2374-8265.11209)
Supplement: Supplementary file 1 — Door Charts.docxSimulated EHR.pptxSession Logistics.docxSP Scripts and Instructions.docxPreclass Student Notes.docxPerspectives From Faculty on EHR.mp4Faculty Instructions.docxEHR Skills - Good Example.mp4EHR Skills - Bad Example.mp4Learner Evaluation.docx [file mep_2374-8265.11209-s001.zip › G. Faculty Instructions.docx]

**Doctor-Patient Relationship (DPR) & the Electronic Health Record (EHR):**

**Faculty Instructions**

**Session overview**

| **Time** | **Activity** |
| --- | --- |
| First 15 minutes | Overview of the doctor-patient relationship & interacting with the EHR |
| The next 2 hours, 20 minutes | Simulated “urgent care” clinic: history taking, DPR, & PE practice; approach to “cough” and “dyspnea” |

**Learning objectives:**

By the end of this activity, learners will be able to:

1. Practice communication strategies to effectively navigate the doctor-patient relationship while interacting with the electronic health record (EHR).

2. Verify information from a simulated EHR while obtaining history from a standardized patient during a simulated clinical encounter.

3. Perform additional, focused history taking based on data verified from the EHR.

**Pre-class preparation**:

Be sure to review the pre-class assigned readings, videos, and written assignment instructions for students for this session (listed below):

- Review the student notes, “The Electronic Health Record and the Doctor-Patient Relationship,**”** included as appendix E.
- **Watch the video, “**Perspectives from Experienced Faculty on the EHR and the Practice of Medicine**” included as appendix F.**
- **There are videos that were created as a pre-learn activity only for the students, “EHR Example Video Good,” and “EHR Example Video Bad” (appendices H and I) that are available if you are interested.**

**In-class activities:**

**Part 1: Review of content from pre-class assigned materials**

**Briefly, review with students the important points from their pre-class notes on the “Electronic Medical Record and the Doctor-Patient Relationship” and their reflections on the video, “**Perspectives from Experienced Faculty on the EHR**” in preparation for the Simulated Urgent Care Clinic exercise in which they will participate in part 2 of this lesson. Specifically, prompt students to review:**

- **The conceptual framework from the University of Chicago, HUMAN LEVEL.**
- **Challenges of interacting with the EHR and the patient during an encounter;**
- **Important aspects of positioning oneself, the patient, and the computer in the room;**
- **Moments when it is important to stop looking at the computer screen and instead focus on looking directly at the patient;**
- **“Trust but verify” as described in our faculty perspectives video. In short, all information obtained from the EHR should be verified with the patient, rather than assuming it is correct/complete**

**Part 2: Simulated “Urgent Care Clinic” Exercise**

*NOTE: it is important that this activity begins promptly on time for all student groups, and that all groups stay on schedule as directed.*

The goal of this exercise is for the student to assume the role of the physician in a simulated urgent care clinic and practice the strategies discussed in their pre-class assignments and preceding discussion. This exercise is designed for small groups of 1 faculty and up to 4 students.

**Logistical overview and timing:**

This activity will include a series of 4 patient encounters, so each student in your group should have an opportunity to lead a history and perform a PE with an SP at least once.  Each iteration of the activity will take place as follows:

- **SP encounter** (first 20 minutes): One student will lead each 20-minute encounter with an SP; meanwhile, the faculty and other students will observe the encounter and prepare feedback for the interviewer.  See below for a description of the expected tasks for students to perform during these encounters.
- **Debrief** (next 10 minutes): Following each encounter, there will be a 10-minute debrief period for feedback; involve the observing students in the discussion.
- **Transition time** (next 5 minutes): 5 minutes are allotted prior to the next encounter for the next student interviewer to proceed to their assigned exam room for the next encounter and review the EHR and door chart.

For each case, the interviewing student will proceed to the assigned exam room for the applicable case, according to your group’s assigned room rotation schedule.

It may be helpful to assign particular tasks to each observer, so that they can focus on one or two unique aspects of the encounter (e.g., positioning of individuals/computer screen in the room; nonverbal cues; verification of information found in the EHR; history taking approach for the HPI, etc.).

**SP encounter tasks:** Specific student tasks for these encounters include:

1. **Practice greeting and connecting with the patient.**
2. **Practice using open-ended questions to elicit the history of present illness and characterize the associated symptoms.**
3. **Practice reviewing and updating the past medical, family, and social histories, medications, and allergies utilizing the EHR:**

Note that, for these cases, students will not be in the role of the patient’s primary care physician; therefore, it will be important for them to verify the patient’s pertinent health history, medications, allergies and social history (for the latter, instruct students to focus on tobacco use and the patient’s occupation).

Instruct students to use the information provided in the simulated EHR on the in-room computer as a starting point, and to “trust but verify” the information they find in this EHR with the patient during the interview.

This exercise will work best if the EHR (which is a powerpoint file) is kept in SLIDESHOW mode, and the student simply clicks the applicable links within each slide to navigate within the EHR. If the student accidentally ends the “show,” instruct the student to put the file back into “slideshow” mode.

4. **Perform a physical exam, including vital signs and a targeted physical exam for the complaint.**

Students can determine, using their differential diagnosis for each case, which physical exam maneuvers seem most relevant to determining the source of the patient’s symptoms. For the cases in this set, this should at least include vital signs and a lung exam.

**Debrief**

Immediately following each patient encounter, student interviewers should return to your group’s assigned meeting room, to debrief with you and the other students in your small group. Start by prompting the student interviewer to reflect on the encounter; for example:

- *How did it go?*
- *What did you find most challenging?*
- *What might you do differently next time?*

Next, prompt the observing students in your group to provide feedback to the student interview, based on their observations of the encounter.

Finally, provide your own perspective of the student interviewer’s performance to fill in any gaps not yet mentioned by others in the group. Focus on 1 or 2 key aspects of the student’s performance, including encouragement for tasks s/he performed well. Connect the students’ observations to your own experiences in clinical practice, where applicable.
