# Supplementary material for: Integrating the Electronic Health Record Into Patient Encounters: An Introductory Standardized Patient Exercise for Preclinical Medical Students
Source: MedEdPORTAL. 2022 Jan 5;18:11209. doi: 10.15766/mep_2374-8265.11209 (PMC8727442; doi:10.15766/mep_2374-8265.11209)
Supplement: Supplementary file 1 — Door Charts.docxSimulated EHR.pptxSession Logistics.docxSP Scripts and Instructions.docxPreclass Student Notes.docxPerspectives From Faculty on EHR.mp4Faculty Instructions.docxEHR Skills - Good Example.mp4EHR Skills - Bad Example.mp4Learner Evaluation.docx [file mep_2374-8265.11209-s001.zip › Educational_Summary_Report_11209.pdf]

MedEdPORTAL® | The AAMC Journal of Teaching and Learning Resources

Original Publication

Open Access

Integrating the Electronic Health Record Into Patient Encounters: An Introductory Standardized Patient Exercise for Preclinical Medical Students

Joseph A. Cristiano, MD*, Jennifer M. Jackson, MD, E Shen, PhD, Donna M. Williams, MD, Leslie R. Ellis, MSHPEd, MD

*Corresponding author: jcristia@wakehealth.edu

Abstract

Introduction: Increasingly, use of the electronic health record (EHR) is interwoven into even the most basic patient care tasks. Accordingly, learning how to utilize the EHR during patient encounters is important for medical students as they develop their clinical skills. Existing EHR curricula have focused primarily on doctor-patient relationship skills. We developed a session for our preclinical students on EHR-related doctor-patient relationship skills as well as on using the EHR to verify data and focus one's history taking. **Methods:** We developed student notes, three training videos, four standardized patient (SP) cases, and a simplified, simulated EHR based on these cases. Students reviewed the notes and videos prior to class. During class, students practiced EHR-related communication and data-collection strategies by interviewing an SP while interacting with the simulated EHR. Following each encounter, students received feedback from a small group of peers and faculty. **Results:** Two-hundred eighty-nine second-year medical students participated this session in 2019 and 2020, and 27 (19%, 2019) and 40 (28%, 2020) students, respectively, completed the postsession evaluation. Most respondents rated the SP activity as extremely or quite effective for practicing doctor-patient relationship strategies while interacting with the EHR (89%, 2019; 83%, 2020) and for practicing verification of EHR data during a patient encounter (81%, 2019; 86%, 2020). **Discussion:** This training session was effective for introducing preclinical medical students to fundamental concepts and skills related to incorporating the EHR into patient encounters and offers a low-cost approach to teaching early medical students these important skills.

Keywords
Electronic Health Record, Physician-Patient Relationship, Standardized Patient, Communication Skills, Clinical Skills Assessment/OSCEs

Educational Objectives

By the end of this activity, learners will be able to:

1. Practice communication strategies to effectively navigate the doctor-patient relationship while interacting with the electronic health record (EHR).
2. Verify information from a simulated EHR while obtaining history from a standardized patient during a simulated clinical encounter.
3. Perform additional, focused history taking based on data verified from the EHR.

Citation:
Cristiano JA, Jackson JM, Shen E, Williams DM, Ellis LR. Integrating the electronic health record into patient encounters: an introductory standardized patient exercise for preclinical medical students. *MedEdPORTAL*. 2022;18:11209.
https://doi.org/10.15766/mep_2374-8265.11209

Introduction

The Health Information Technology for Economic and Clinical Health Act of 2009[1] and the Patient Protection and Affordable Care Act of 2010[2] provided a powerful catalyst for the rapid proliferation of technologically advanced electronic health record (EHR) systems that are now commonplace throughout nearly all modern-day US health care institutions.[3] Most physicians now report using an EHR to fulfill an increasing number of the core clinical duties of the practice of medicine.[4] Effective incorporation of the EHR into a patient encounter is a necessity for maintaining the doctor-patient relationship. Accordingly, best-practice strategies have emerged to enhance communication and relationship building while constructively incorporating the EHR into the clinical encounter.[5-8]

This transformation in health care delivery has generated a great need within undergraduate medical education for curricular enhancements and updates to address the myriad of new EHR-specific skill sets essential to modern-day patient care.[3,9] Curricular development for incorporating the EHR into the

Copyright © 2022 Cristiano et al. This is an open-access publication distributed under the terms of the Creative Commons Attribution license.

doctor-patient relationship is an area of growing attention, and several educational interventions have been described in the literature.[7,10-13] These studies demonstrate that students perform better after EHR-specific communication training for relationship building and suggest that these skills are necessary and teachable.

Although less proliferative, there has been some progress in teaching trainees complex electronic data-gathering skills and documentation skills.[11,13-19] This includes strategies to balance information gathered in advance of the visit from the EHR with information gathered directly from the patient. Zavodnick and Kouvatsos recently published a workshop on some of these skills, but it was designed for fourth-year medical students.[15] Providing instruction for preclinical students on strategies for trusting information originating from the EHR but also verifying it with the patient can help them develop constructive habits early on that support their ability to collect an accurate account of a patient's history.

Combining skills in use of the EHR for encouraging relationship building and effective communication with those EHR skills for data gathering and for focusing the patient encounter, we strongly believe that there is an emerging comprehensive skill set. This skill set aligns with those basic clinical skills currently taught early in medical education in gathering a history and performing an exam. Identifying and introducing those complementary EHR skills can encourage early skill development. Additionally, setting professional expectations early in trainees' educational journey could also mitigate the impact of the hidden curriculum they may encounter in their clerkships when observing providers who neglect to confirm key historical data from the EHR and inadvertently perpetuate mistakes and errors in their history gathering and documentation.[20-22]

This publication builds on existing educational resources on this topic, which have focused primarily on the doctor-patient relationship and patient-centered communication skills necessary for incorporation of the EHR into the clinical encounter. LoSassso and colleagues[12] and Lee and colleagues[7] have developed similar conceptual frameworks and assessment instruments for use in teaching and evaluating EHR-specific communication strategies. Our educational activity incorporates the HUMAN LEVEL mnemonic, as developed by Lee and colleagues, as a comprehensive conceptual framework.[12] Complementing this available instructional content, we have created videos that reinforce these concepts as well as introduce our students to specific strategies in data gathering for conducting a focused history and physical exam for a complaint-driven visit.

Here, we describe our approach to introducing EHR-related doctor-patient relationship skills along with data-gathering concepts for our preclinical medical students. This approach features a set of preclass instructional content, preclass demonstration videos, and in-class practice activities including a series of standardized patient (SP) encounters in which students evaluate patients in a simulated ambulatory acute care setting. These encounters function as an introductory application of the communication strategies for maintaining the doctor-patient relationship while also simultaneously practicing verification of EHR data with the patient during history taking. This session was designed to promote learning based on the principles of deliberate practice and Kolb's experiential learning cycle. Through the SP encounter series, learners have an opportunity to practice applying newly learned concepts and skills in a realistic clinical context, followed immediately by performance feedback from peers and faculty.[23] Over the course of the session, learners participate in the concrete experience, reflective observation, abstract conceptualization, and active experimentation stages of Kolb's cycle, thereby reinforcing knowledge and skill development.[24] This session is complementary to our EHR observed structured clinical exam station that assesses students' EHR skill set during their third-year clerkships.[25,26]

Methods

Educational Context

This activity was developed for use within the clinical skills course taught longitudinally throughout our 18-month preclerkship curriculum; that course introduced the fundamental communication skills for doctor-patient relationship building, history taking, physical examination, clinical reasoning, oral presentation, and written documentation skills. The course was delivered in small groups led by clinician faculty and was designed based on deliberative practice principles incorporating direct observation of iterative student practice with immediate formative feedback through real and simulated patient encounters.

The lesson described in this resource was implemented approximately 12 months into our students' 18-month preclinical curriculum. At this time in their curriculum, students were also participating in the pulmonology course; therefore, the SP cases designed for this session focused on respiratory complaints. The cases were designed by course directors with careful consideration of the array of clinical skills previously introduced, practiced, and assessed during the first 12 months of the course. The EHR-specific skills were the only ones not previously introduced or practiced, and it was the intent of this experience

Copyright © 2022 Cristiano et al. This is an open-access publication distributed under the terms of the Creative Commons Attribution license.

to introduce these new skills while using those other skills since much of the challenge of the EHR skills was their simultaneous incorporation with the array of other basic clinical skills.

Prior to this lesson, these second-year medical students had been previously introduced to, had practiced, and had been assessed on basic doctor-patient relationship communication skills, comprehensive history gathering, and basic physical exam maneuvers. Students had also begun practicing hypothesis-driven data gathering for several common chief complaints. We felt that it was important to introduce these EHR-related skills before students transitioned to the clerkship years but that it was also important to do so after students had learned how to perform essential patient-encounter skills. Specific knowledge or skills related to the EHR were not required for students' participation in this learning activity.

Activity Preparation

Space preparation: This session was conducted in our medical school's simulation center and required the use of six small-group meeting rooms and 12 clinical exam rooms on each of 3 afternoons to accommodate a class of 140-145 students. Before the session began, we placed on the outside of the door to each exam room a copy of the patient's simulated chart (Appendix A), which included the patient's name, age, chief complaint, and set of vital signs. We also loaded a copy of the simulated EHR for each case (Appendix B) on the computer in each exam room.

SP recruitment: Based on our logistical plan for this activity (Appendix C), our SP program manager recruited 12 SPs to participate in this class session each afternoon. We sent instructions and case scripts (Appendix D) to the SPs in advance of the class session; in-person SP training was not performed for this specific case but had been performed previously for other lessons.

Preclass Assignments and Resources

We assigned preclass materials for students and faculty to review in advance of the session, including a brief reading introducing the key concepts of the doctor-patient relationship and the EHR with selected citations and use of the HUMAN LEVEL mnemonic[12] (Appendix E) and a video of interviews with five practicing academic physicians who shared perspectives regarding data gathering, EHR-focused interviewing, and electronic documentation (Appendix F). We also sent faculty a set of instructions for the lesson plan for the session (Appendix G). Additional optional resources offered to students included videos of simulated encounters exemplifying both good and bad EHR skills performances (Appendices H and I).

In-Class Activities

This lesson was delivered over a class session lasting 2 hours and 35 minutes. To begin class, faculty in each small group reviewed the preclass assigned materials during a 15-minute facilitated discussion of their concepts. We encouraged faculty to share their own reflections on and experiences with this skill set during the discussion.

Following this introductory discussion, all small groups participated in a series of four 20-minute SP encounters. Student small groups were split in half, into mini-groups (three to four students with one faculty each), so that, over the course of the activity, all students had an opportunity to practice these skills during at least one SP encounter. The context for each of these SP visits was an acute care visit for a respiratory chief complaint, with the expectation that students would maintain traditional doctor-patient relationship skills adapted for the EHR and conduct a complaint-driven clinical encounter with the added expectation for the student to verify structured data elements in the EHR with the patient. During each SP encounter, one student interviewed the SP while the other students and faculty observed (in 2019, mini-groups observed the encounter from the back of the exam room; in 2020, mini-groups observed the encounter from a small-group meeting room through cameras installed in the exam rooms using LearningSpace software, to permit social distancing).

We instructed students to refer to the simulated EHR on the computer in their room throughout the SP encounter. Specifically, students were instructed to practice the doctor-patient relationship skills related to the EHR (i.e., HUMAN LEVEL) and to verify the information located in each section of the EHR as they obtained a history from the SP.

At the conclusion of each SP encounter, faculty facilitated a 10-minute debrief, during which the student interviewer was prompted to reflect on their performance and the faculty, SP, and observing students provided feedback to the student interviewer.

After class, students wrote a note based on their SP encounter. Faculty reviewed these notes and provided students with individualized formative feedback on their documentation.

Program Evaluation

After the class session, we sent students a voluntary online survey on these EHR skills to explore their attitudes toward the importance of the activity's learning content and to assess their evaluation of the activity's effectiveness for meeting its learning objectives (Appendix J).

Copyright © 2022 Cristiano et al. This is an open-access publication distributed under the terms of the Creative Commons Attribution license.

This study was reviewed and approved by the Wake Forest School of Medicine Institutional Review Board (IB00059744).

Results

The lesson was delivered in August 2019 and August 2020 at the Wake Forest School of Medicine, and 289 second-year medical students participated in this activity over the 2 years. Twenty-seven students (19%) completed the postsession survey in 2019, and 40 students (28%) completed the postsession survey in 2020.

The mean student ratings of the overall effectiveness of this learning activity were 7.9 and 8.1 out of 10 in 2019 and 2020, respectively (1 = *poor,* 10 = *excellent*). Most respondents (89%, 2019; 86%, 2020) rated the class session as extremely or quite relevant. Nearly all respondents assessed the small-group format as appropriate for learning the content (100%, 2019; 95%, 2020). Most respondents agreed or strongly agreed that the preparatory time required to review the assigned preclass materials was manageable (81%, 2019; 76%, 2020). Most respondents rated the SP exercise as extremely or quite effective for practicing strategies for maintaining the doctor-patient relationship while interacting with the EHR (89%, 2019; 83%, 2020) and verifying data in the EHR during the patient encounter (81%, 2019; 86%, 2020). Most respondents also assessed the SP encounters as effective for understanding the challenges of navigating the doctor-patient relationship while interacting with the EHR (78%, 2019; 76% 2020).

Respondents' narrative comments were positive overall. A consistent theme was that the students valued the opportunity to practice with a simulated EHR, through which they recognized the challenges of its use when conducting a patient visit. Student comments also recognized that the simulated EHR interface was simpler than the more complex EHR used in clinical practice. Some respondents expressed appreciation for the simpler format while others voiced interest in a higher-fidelity simulated EHR. Other respondents also expressed interest in being able to preview the electronic chart prior to the visit.

Respondents' constructive criticism thematically related to time management of the session, with some respondents feeling rushed. A few students suggested this could be mitigated by having the EHR available before and after the SP session. One respondent suggested it could be helpful for the SP to have a grading rubric for more specific feedback on learner performance of communication skills from the patient's perspective.

Discussion

In this training session, we have incorporated the emerging fund of curricular innovations related to EHR training and the doctor-patient relationship.[7,10-13,27] Including EHR training early in students' educational journey introduces them to emerging best practices in EHR use, as well as encouraging early adoption of doctor-patient relationship skills when using the EHR.[8] The session also incorporates instruction on appropriately balancing history gathered from the EHR and from the patient. Without this explicit instruction, learners may develop inappropriate data-gathering and documentation habits rather than systematically applying data-checking strategies such as "trust but verify" (i.e., confirming information that is gathered from the EHR with the patient). In one survey of 123 third-year medical students, 31% believed it was appropriate to copy and paste from others' clinical documentation, including the past medical, social, and family history as well as medications.[28] By proactively training students on the crucial importance of verifying information from the EHR as they learn fundamental history-gathering skills, we couple data-gathering skills to important concepts of professionalism, such as ensuring the accuracy and completeness of data collection and subsequent reporting. By incorporating learner practice of multiple EHR-related skills, our resource represents a contribution to the medical education literature.

Students' feedback on this lesson was overall very positive and indicated that they felt this instructional approach was effective at addressing the activity's learning objectives. The lesson was designed to provide instruction on the learning objectives through the use of curricular scaffolding. We incorporated this activity at a point in students' clinical skills curriculum when they had begun honing their fundamental doctor-patient relationship and history-taking skills so that they could then approximate those skill sets to the related EHR proficiencies. We intentionally built a very simplified simulated EHR so that students would not be distracted by the inherent complexities of using a fully functional EHR platform and could instead focus on practicing the doctor-patient relationship and data-gathering aspects of the patient encounter. By utilizing this simplistic design, we were able to introduce novice students to the demands of multitasking during a patient visit without overwhelming them. We also implemented the lesson without using a sophisticated or institution-specific EHR simulation, increasing its generalizability for other educators.

The lesson was designed to incorporate the Kolb experiential learning model by utilizing simulation-based practice through SP encounters and a simulated EHR. Students practiced the

Copyright © 2022 Cristiano et al. This is an open-access publication distributed under the terms of the Creative Commons Attribution license.

newly introduced EHR skills (concrete experience), then actively reflected on the experience and sought feedback from their peers and faculty coaches (reflective observation and abstract conceptualization). Since the logistical model we used allowed the four students in a mini-group to reflect on each other's performance, learners who practiced these skills later in the SP encounter rotation were able to incorporate observations and feedback from other students' prior experiences into their own performance (active experimentation).

A significant limitation of this study is that the evaluation of the effectiveness of the lesson was quite limited considering the very low response rate to our learner evaluation survey. We speculate that the response rate in the first year was particularly low because several weeks had elapsed between the activity and the survey being sent. In the second year, the survey was sent promptly after the experience, and the response rate was similar to our experiences in other studies at our institution. We also acknowledge that our outcomes were limited to student perceptions of the learning activity rather than including learner performance data.

In addition, we did not include a formative assessment tool to systematically assess students' performance of the EHR-related skills during the activity. We felt that clinical coaches who facilitated routinely would be capable of giving actionable feedback interweaved with their own individualized experiences. However, use of such a tool would aid in providing standardized feedback to students during the debrief.

Future directions include developing a formative feedback tool that will enhance actionable and specific feedback for our learners and devising additional practice activities to provide students with opportunities to practice and receive feedback on these skills later in the curriculum, so that the skills and concepts continue to be reinforced.

Appendices

A. Door Charts.docx
B. Simulated EHR.pptx
C. Session Logistics.docx
D. SP Scripts and Instructions.docx
E. Preclass Student Notes.docx
F. Perspectives From Faculty on EHR.mp4
G. Faculty Instructions.docx
H. EHR Skills - Good Example.mp4
I. EHR Skills - Bad Example.mp4
J. Learner Evaluation.docx

All appendices are peer reviewed as integral parts of the Original Publication.

Joseph A. Cristiano, MD: Assistant Professor, Department of Internal Medicine, Section of General Internal Medicine, Wake Forest School of Medicine; ORCID: https://orcid.org/0000-0003-1388-5867

Jennifer M. Jackson, MD: Associate Professor, Department of Pediatrics, Section of Pediatric Hospital Medicine, Wake Forest School of Medicine; ORCID: https://orcid.org/0000-0002-0179-7379

E Shen, PhD: Assistant Professor, Department of Internal Medicine, Section of General Internal Medicine, Wake Forest School of Medicine; ORCID: https://orcid.org/0000-0001-6751-5445

Donna M. Williams, MD: Associate Professor, Department of Internal Medicine, Section of General Internal Medicine, Wake Forest School of Medicine; ORCID: https://orcid.org/0000-0003-2364-2199

Leslie R. Ellis, MSHPEd, MD: Associate Professor, Department of Internal Medicine, Section on Hematology and Oncology, Wake Forest School of Medicine; ORCID: https://orcid.org/0000-0003-0459-7951

Disclosures
None to report.

Funding/Support
None to report.

Ethical Approval
The Wake Forest School of Medicine Institutional Review Board approved this project.

References

1. Health Information Technology for Economic and Clinical Health Act, Pub L No. 111-5, 123 Stat 227 (2009).
2. Patient Protection and Affordable Care Act, *Pub L No. 111-48*, 124 Stat 119 (2010).
3. Blumenthal D. Wiring the health system—origins and provisions of a new federal program. *N Engl J Med*. 2011;365(24): 2323-2329. https://doi.org/10.1056/NEJMsr1110507
4. Norton PT, Rodriguez HP, Shortell SM, Lewis VA. Organizational influences on healthcare system adoption and use of advanced health information technology capabilities. *Am J Manag Care*. 2019;25(1):e21-e25.
5. Johnson KB, Serwint JR, Fagan LA, Thompson RE, Wilson MEH, Roter D. Computer-based documentation: effects on parent-provider communication during pediatric health

Copyright © 2022 Cristiano et al. This is an open-access publication distributed under the terms of the Creative Commons Attribution license.

maintenance encounters. *Pediatrics*. 2008;122(3):590-598. https://doi.org/10.1542/peds.2007-3005

6. Silverman H, Ho YX, Kaib S, et al. A novel approach to supporting relationship-centered care through electronic health record ergonomic training in preclerkship medical education. *Acad Med*. 2014;89(9):1230-1234. https://doi.org/10.1097/ACM.0000000000000297
7. LoSasso AA, Lamberton CE, Sammon M, et al. Enhancing student empathetic engagement, history-taking, and communication skills during electronic medical record use in patient care. *Acad Med.* 2017;92(7):1022-1027. https://doi.org/10.1097/ACM.0000000000001476
8. Duke P, Frankel RM, Reis S. How to integrate the electronic health record and patient-centered communication into the medical visit: a skills-based approach. *Teach Learn Med*. 2013; 25(4):358-365. https://doi.org/10.1080/10401334.2013.827981
9. Tierney MJ, Pageler NM, Kahana M, Pantaleoni JL, Longhurst CA. Medical education in the electronic medical record (EMR) era: benefits, challenges, and future directions. *Acad Med*. 2013; 88(6):748-752. https://doi.org/10.1097/ACM.0b013e3182905ceb
10. Alkureishi MA, Lee WW, Lyons M, Wroblewski K, Farnan JM, Arora VM. Electronic-Clinical Evaluation Exercise (e-CEX): a new patient-centered EHR use tool. *Patient Educ Couns*. 2018;101(3):481-489. https://doi.org/10.1016/j.pec.2017.10.005
11. Biagioli FE, Elliot DL, Palmer RT, et al. The electronic health record objective structured clinical examination: assessing student competency in patient interactions while using the electronic health record. *Acad Med.* 2017;92(1):87-91. https://doi.org/10.1097/ACM.0000000000001276
12. Lee WW, Alkureishi ML, Wroblewski KE, Farnan JM, Arora VM. Incorporating the human touch: piloting a curriculum for patient-centered electronic health record use. *Med Educ Online*. 2017;22(1):1396171. https://doi.org/10.1080/10872981.2017.1396171
13. Morrow JB, Dobbie AE, Jenkins C, Long R, Mihalic A, Wagner J. First-year medical students can demonstrate EHR-specific communication skills: a control-group study. *Fam Med*. 2009;41(1):28-33.
14. Milano CE, Hardman JA, Plesiu A, Rdesinski RE, Biagioli FE. Simulated electronic health record (Sim-EHR) curriculum: teaching EHR skills and use of the EHR for disease management and prevention. *Acad Med*. 2014;89(3):399-403. https://doi.org/10.1097/ACM.0000000000000149
15. Zavodnick J, Kouvatsos T. Electronic health record skills workshop for medical students. *MedEdPORTAL*. 2019;15:10849. https://doi.org/10.15766/mep_2374-8265.10849
16. Bierman JA, Hufmeyer KK, Liss DT, Weaver AC, Heiman HL. Promoting responsible electronic documentation: validity evidence for a checklist to assess progress notes in the electronic health record. *Teach Learn Med*. 2017;29(4):420-432. https://doi.org/10.1080/10401334.2017.1303385
17. March CA, Scholl G, Dversdal RK, et al. Use of electronic health record simulation to understand the accuracy of intern progress notes. *J Grad Med Educ*. 2016;8(2):237-240. https://doi.org/10.4300/JGME-D-15-00201.1
18. Stetson PD, Bakken S, Wrenn JO, Siegler EL. Assessing electronic note quality using the Physician Documentation Quality Instrument (PDQI-9). *Appl Clin Inform*. 2012;3(2):164-174. https://doi.org/10.4338/ACI-2011-11-RA-0070
19. Stetson PD, Morrison FP, Bakken S, Johnson SB; eNote Research Team. Preliminary development of the Physician Documentation Quality Instrument. *J Am Med Inform Assoc*. 2008;15(4):534-541. https://doi.org/10.1197/jamia.M2404
20. Schenarts PJ, Schenarts KD. Educational impact of the electronic medical record. *J Surg Educ*. 2012;69(1):105-112. https://doi.org/10.1016/j.jsurg.2011.10.008
21. Wrenn JO, Stein DM, Bakken S, Stetson PD. Quantifying clinical narrative redundancy in an electronic health record. *J Am Med Inform Assoc*. 2010;17(1):49-53. https://doi.org/10.1197/jamia.M3390
22. Lehmann LS, Sulmasy LS, Desai S; ACP Ethics, Professionalism and Human Rights Committee. Hidden curricula, ethics, and professionalism: optimizing clinical learning environments in becoming and being a physician: a position paper of the American College of Physicians. *Ann Intern Med*. 2018;168(7):506-508. https://doi.org/10.7326/M17-2058
23. Ericsson KA. Deliberate practice and the acquisition and maintenance of expert performance in medicine and related domains. *Acad Med*. 2004;79(10)(suppl):S70-S81. https://doi.org/10.1097/00001888-200410001-00022
24. Kolb DA. *Experiential Learning: Experience as the Source of Learning and Development*. Prentice-Hall; 1984.
25. Ellis LR, Cristiano J, Jackson JM, Shen E. Navigating doctor-patient relationships in the EHR age. *Med Educ*. 2020;54(5):441. https://doi.org/10.1111/medu.14105
26. Shen E, Cristiano JA, Ellis LR. The electronic health record objective structured clinical examination station: assessing student competency in patient notes and patient interaction. *MedEdPORTAL*. 2020;16:10998. https://doi.org/10.15766/mep_2374-8265.10998
27. Lee WW, Alkureishi ML, Arora VM. Integrating longitudinal training to promote competency in patient interactions while using the electronic health record. *Acad Med*. 2017;92(2):141-142. https://doi.org/10.1097/ACM.0000000000001522
28. Heiman HL, Rasminsky S, Bierman JA, et al. Medical students' observations, practices, and attitudes regarding electronic health record documentation. *Teach Learn Med*. 2014;26(1):49-55. https://doi.org/10.1080/10401334.2013.857337

Received: March 24, 2021
Accepted: September 25, 2021
Published: January 5, 2022

Copyright © 2022 Cristiano et al. This is an open-access publication distributed under the terms of the Creative Commons Attribution license.
